# 

**Published:** 1901-10-05

**Authors:** 


### The Hospital

A JOURNAL OF

THE MEDICAL SCIENCES AND HOSPITAL ADMINISTRATION,

INCLUDING

### A SPECIAL SECTION FOR NURSES.

IN TWO VOLUMES ANNUALLY.

EDITED BY

SIR HENRY BURDETT, K.C.B..

AND

SOLOMON C. SMITH, M.D.

67

LIBRARY

SURGEON GENERAL'S OFFICE

OCT.-2--1902

180507.

SGO

VOLUME XXXI.

OCTOBER 1901 TO MARCH 1902.

London:

PUBLISHED BY THE SCIENTIFIC PRESS (LIMITED), AT THE HOSPITAL BUILDING,
28 & 29 SOUTHAMPTON STREET, STRAND, W.C.

MDCCCCII.

W1
H069X

### INDEX TO VOL. XXXI., 1902.

*** Articles under the general heading *Hospital Clinics and Medical Progress* are distinguished by the initial (C.) for *Hospital Clinics*, and by (P.) for the rest of the series. (W.) distinguishes the *Institutional Workshop* articles, and (A.) the *Annotations.*

Abuse of Prescriptions, The, 432
Addenbrooke's Hospital (W.), 346
Adenoids (P.), 237
Adenoids in Infancy (C.), 357
Adenoids, When to Remove (C.), 95
Advantage of being an Invalid, The (A.), 213
"After-Cataract," Treatment of (P.), 168
Age and Activity, 75
Alæ, Nasi, Collapse of the (P.), 289
Albuminuria in Pregnancy, Treatment of (C.), 237
Albumosuria (P.), 116
Alcohol in the Causation of Disease (C.), 235
Alcohol and the Race (A.), 231
Alcoholism, The Heart in (P.), 32
Aliens in the East End (A.), 319
Alcoholism to Suicide, The Relation of (C.), 404
Anæmia, Pernicious (P.), 134; Splenic (P.), 186
Anæsthesia in the Ear, Production of (P.), 360
Anæsthetics, Progress in (P.), 287
Ankylostomiasis (C.), 49
Antiseptics on the Battlefield (C.), 163
Anti-typhoid Inoculation (C.), 96
Anti-vaccinators, 44
Anti-vaccinist and Smallpox Ships, The (A.), 319
Antrum of Highmore (P.), 307
Aorta, Aneurysm of the (C.), 97
Aortic Disease in Childhood, Acquired (P.), 202
Appendicitis (P.), 340
Appendix, What to do with the (C.), 373
Army Medical Service, The (A.), 145
Army Medical Service Reform (A.), 45
Army Medical Service, Reorganisation of the (C.), 5
Arterio-Sclerosis (P.), 101
Arthritis, Rheumatoid (C.), 63
Asylum Treatment and "Medical Relief" (A.), 195
Aural Polypi (P.), 324
Auscultation of the Descending Aorta (P.), 327
Bacillus, the Filiform, and Cancer of the Stomach (P.), 82
Bacteriology (in Dermatology) (P.), 52
Bacteriology, Progress in (P.), 16, 35, 82, 270, 376
Bad Arms (A.), 247
Beef Tea and Meat Preparations (W.), 294, 312, 329, 346
Belgrave Hospital for Children, Clapham Road (W.), 38
Bengal, The Charitable Dispensaries of (W.), 294
Beri-Beri, The Lessons of (C.), 148
Bird-Lime (W.), 104
Bladder, Immediate Suture of the (P.), 167
Bladder, Tumours of the (P.), 167
Blood, Histology of the (P.), 186
Blood, Progress in Diseases of the (P.), 134, 186
Boarding Out (A), 385
Boer Concentration Camps (A.), 3
Boiled *versus* Unboiled Milk (P.), 218
Bolingbroke Hospital, Wandsworth (W.), 155
Book World (*Books, Magazines, and Pamphlets Reviewed*)
Addyman. Practical X-Ray Work, 37
Barr. Manual of Diseases of the Ear, 54
Broadbent. *See Squire.*
Cabot. Physical Diagnosis of Diseases of the Chest, 37
Carpenter. Golden Rules for Diseases of Children, 137
Choksy. Report on the Treatment of Plague, 345
Colyer. *See Smale.*
Crile. Experimental and Clinical Research into certain Problems Relating to Surgical Operations, 310
De Meric. Syphilis and other Venereal Diseases, 257
Donald. The London Manual for 1902, 292
Donkin. Smoke and its Diminution, 292
Doyle. The War in South Africa, 310
Ellis. The Criminal, 171
Finsen (trans. by Sequeira). Phototherapy, 120
First Aid Diagrams for the Use of Lecturers, 171
Frick. Liquid Air and its Application, 27[illegible]
Furneaux. Elementary Practical Hygiene, 257
Geddes and Thompson. The Evolution of Sex, 222
Giles. Menstruation and its Disorders, 222
Goodsall and Miles. Diseases of the Anus and Rectum, 86
Gould and Pyle. Pocket Cyclopædia of Medicine, 292
Gray (edited by Pick and Howden). Anatomy, 70
Haig. Diet and Food, 171
Harrison. Some Retrospects and Prospects in Surgery, 54
Howden. *See Gray.*
Jennings. On the Cure of Morphia Heart, 204
Journal of Obstetrics and Gynæcology of the British Empire, 274
Lake. International Directory of Laryngologists and Otologists, 102
Mayer. *See Oppenheim*
Mayo-Robson and Moyniham. Diseases of the Stomach, 70
Miles. *See Goodsall*
Morris. Surgical Diseases of the Kidney and Ureter, 102
Moyniham. *See Mayo-Robson*
Nall. Aids to Obstetrics, 222
Oldfield. The Penalty of Death, 19
Oppenheim (trans. by Mayer). Diseases of the Nervous System, 19
Pick. *See Gray*
Poland. Retrospect of Surgery (Hunterian Oration), 204
Power. Atlas of the Anatomy and Physiology of the Child, 37
Probyn-Williams Practical Guide to the Administration of Anæsthetics, 204
Pyle. *See Gould*
Quilter. What's What, 154
Ramsay. The Scientific Roll and Magazine of Systematised Notes, Vol. I., 120
Reid. Alcoholism, 136
Reynolds-Ball. Jerusalem: A Practical Guide, 171
Roche (edited by). The Imperial Health Manual, 345
Sequeira. *See Finsen.*
Sherrington. Lecture on Physiology for Teachers, 54
Skirving. Our Army in South Africa, 120
Smale and Colyer. Diseases and Injuries of the Teeth, 154
Squire (Introd. by Broadbent). Essays on Consumption, 136
Thomson. *See Geddes*
Thomson. A Treatise of Plague, 54
Timberg. Home Exercises, 310
Treves. Surgical and Applied Anatomy, 19
Van Ryn. The Composition of Dutch Butter, 345
Waldo. Golden Rules of Hygiene, 19
Wanklyn. Arsenic, 70
Whitla. Dictionary of Treatment, 120
Williams. Uterine Tumours, 86
Bright's Disease, The Cure of, by Operation (C.) 250
Bright's Disease, Chronic, Treatment of (C.), 197
Bronchitis, Chronic (C.), 48
Bronchocele, Epidemic (C.), 63
Browne's (Mr. Buckston) Views (C.), 356
Burns, The Treatment of (C.), 30
Cæsarian Section (C.), 47
Calomel as a Panacea (C.), 48
Cancer (P.), 35, 341
Cancer Cures, More (C.), 323
Cancer, Inoperable (C.), 65
Cancer, Progress in (P.), 406
Cancer of the Pylorus, The Early Diagnosis of (C.), 130
Cancer, Salt a Suggested Cause of (C.), 183
Cancer of the Stomach and the Filiform Bacillus (P.), 82
Cancer, Theories about (A.), 301
Cancer, X-rays in (C.), 183
"Canvassing," 178
Carbonate of Creasote in Pneumonia (C.), 129
Carcinoma of Stomach (P.), 184
Catheters, The Sterilisation of (P.), 168
Celluloid, Inflammable (C.), 372
Central Public-House Trust Association, The, 170
Central School of Medicine for London, A, 212
Cerebral Surgery, Progress in (P.), 200
Cerebro-Spinal Meningitis (P.), 82
Cervical Sympathetic, Paralysis of the (P.), 151
Changing Fashions in Medical Treatment, 281
Cheap Doctor, The, 245
Children's Homes, The Registration and Inspection of (W.), 425
Chill as a Cause of Disease (A.), 3
Chloroform, Deaths from (C.), 270
Chorea (P.), 151
Chorea of Pregnancy (P.), 118
Chorea: Rheumatism of the Brain (C.), 64
Chromidrosis (C.), 403
Circulatory System in Incurably Deaf Children, 249
Circumcision for the Relief of Acne (C.), 339
Clay *versus* Gravel, 351
Coffee-drinking Boers, The (A.), 265
Conscience Clause in the Vaccination Act, The (A.), 3
Conscientious Objector, The (433)
Constitutio Lymphatica (P.), 135
Consultation and Consultants, 229
Consumption, Early Home Treatment of (C.), 181
Consumptive Cases in Convalescent and other Homes (W.) 157
Convalescence at the Seaside, 431
Co-operative Research, 333
Coroner, Informing the (A.), 265
"Covering," The Limits of (A.), 335
"Crabbed Age and Youth," 194
Curvature of the Spine, Lateral Management of (C.) 6
Cystitis (P.), 82
Cystoscopy (P.) 153
Cysts, Intraligamentous (P.), 326
David Lewis Hospital, Liverpool, The, 442
Defective Union in Fractures (C.), 181
Delirium, Acute Febrile with Hallucinations (P.), 151
Delusions (P.), 271
Dermatitis (P.), 68
Dermatology, Progress in (P.), 52, 67
Diabetes, Progress in (P.), 438
Diathesis, Sir Dyce Duckworth on (C.), 114
Digestive Organs, Progress in Disease of the (P.), 184, 221, 340, 437
Digitalis (P.), 327
Dilatation, Acute, of the Heart (P.), 15
Dilatation, Acute, of the Stomach (P.), 438
Diphtheria (P.), 253, 376
Diphtheria, Bacteriological (C.), 419
Dispensaries and Charitable Institutions of the Punjab (W.), 172
Dispensaries and Hospitals (A.), 369
Doctors, Midwives, and Patients, 435
Does Club Practice Pay? 93
Domestic Medicine in South Africa, 126
Dysentery (P.), 340
Dysentery, English and South African (C.), 12
Dysmenorrhœa, Spasmodic, Clinical Lecture on (C.) 371
Ear, Foreign Bodies in the (P.), 325
East Anglian Sanatorium for Consumptives at Broomfield (W.), 188
East London Hospital for Children (W.), 411
Ectopic Gestation (P.), 326
Eczema (P.), 53, 67
Editor's Letter Box, 41, 244, 261, 331, 448
Electric Hot-water Bottle, An (C.), 150
Electric Railways, 246
Electricity in Medicine (A.), 111
Electrocution of Czolgosz (C.), 150
Empyæma (P.), 376
Endocarditis, Acute, To arrest (C.), 49
Enema Rashes (C.), 269
Engaging a Doctor (A.), 27
Enteric Fever (P.), 270
Enteric Fever, Inoculation for (C.), 269
Epistaxis (P.), 290
Epistaxis in Rheumatism in Children (C.), 374
Exercises for Weak Feet (C.), 131
Experiments on Hospital Patients (P.), 87
Faith Healing and Small pox (A.), 283
"Farthing Land" (A.), 385
"Fee, A, Will be Paid by the Company" (A.), 301
Fevers, Progress in (P.), 312, 357
Fœtus and Pelvis, The Relation between (C.), 436
Following Midwives (A.), 179
Food Preservatives (A), 161
Foot and Mouth Disease, Reappearance of, 103
Foreign Bodies in the Ear (P.), 325
Foreign Bodies in the Nose (C.), 183
Forsythe *versus* Law, 383
"Fourth Disease" (C.), 318
Fractured Ribs in the Insane (A.), 127
French Hospital, The (W.), 430
Freyer, Dr., on his Operation (C.), 356
Gall-stones, The Frequency of (C.), 165
Garden Cities, 18
Gastralgia (C.), 30
Gastric Hæmorrhage, Concealed (P.), 221

Gastric Tetany (P.), 221
Gastric Ulcer (P.), 437
Gastric Ulcer, Feeding in (O.), 388, 405
Gastritis, Phlegmonous (O.), 304
Gelatine as a Hæmostatic (P.), 187, 309
General Medical Council, The (W.), 173
General Medical Council Election (A.), 111, 143
Genito-Urinary Diseases (P.), 33
Glances at the Hospitals (W.), 41, 56, 72, 88, 121, 140, 174, 227, 243, 261, 313, 331, 348, 365
Gleet, or Chronic Gonorrhœa (P.), 34
Gonorrhœa, Acute (P.), 33
Guy's Hospital (W.), 278
Gynæcology, Progress in (P.), 325
Hæmostatic, Gelatine as a (P.), 187
Hair Ball, A, removed from the Stomach (C.), 306
Heart and Circulation, Diseases of the (P.), 15, 32, 252, 308, 327
Heart Disease in Children (O.), 323
Heart Disease, Surgery in (P.), 339
Heart Murmurs, The Influence of Posture on (O.), 420
Heart, The Senile (P.), 327
Hemiplegia (O.), 64
Home Hospitals Association, The (W.), 426
Hospital Building "While you Wait" (A.), 195
Hospital Expenditure (W.), 378
Hospital Festivals, Meetings, etc., 72, 107, 174, 189, 208, 277, 295, 331, 363, 379, 391
Hospital for the Insane (A.), 352
Hospital Library and Charities Bureau (W.), 20
Hospital Rating (A.), 401
Hospital Saturday Fund (W.), 242, 443
Hospital Scandal, an Amazing (A.), 385
Hospitals, American, an Englishman's Account of Some (O.), 305
Hospitals Association, The (W.), 87
Hospitals with Special Acts of Parliament, 367
"Housing" Question, The, 282
Housing of the Working Classes, The (P.), 220
How to Examine the Mouth of an Infant (O.), 149
Hydronephrosis, Congenital (P.), 440
Hydrophobia, Diagnosis of, by Rapid Examination (O.), 421
Hydrophobia, The Prevention of, 137
Hyperplasia, Sclerotic (P.), 237
Hyperpyrexia (P.), 133
Ichthyol in Skin Diseases (O.), 337
Incarnation of Man, The (A.), 319
Inebriates (O.), 217
Infection, The By-ways of (O.), 31
Infection, Public Libraries and (A.), 417
Infection, Spread of, from Small-pox Hospitals, 416
Infection and Predisposition, 60
Infection, Special Modes of (A.), 283
Infectious Colds (A.), 45
Insane, General Paralysis of the (P.), 132
Insane, The Story of the, from Year to Year (W.), 65, 89, 107, 122, 141, 175, 209, 227, 243, 259, 297, 315, 349, 365, 381
Insanity among Immigrants (A.), 265
Insanity, The Toxic Origin of (O.), 95
Institutional Notes, 312
Intemperance, The Control of, 263
Intestinal Obstruction (P.), 341
Intra-thoracic Tumour of the Posterior Mediastinum (P.), 327
Intussusception in Infants (O.), 97
Intussusception, The Treatment of (P.), 219
Isolation Hospitals (W.), 348
Jaundice, Obstructive (O.), 285
Jewish Home for Incurables, Tottenham (W.), 138
Kidney, Cystic (P.), 440
Kidney, Diseases of the, Progress in (P.), 85, 101, 116
Kidney, Movable (P.), 99, 422
Kidney, Movable, How to Examine for (O.), 198
Kidney, Movable, Treatment of (O.), 198
Kidney, Tumours of the (P.), 441
Kidneys, Anatomical Association of the (P.), 117
Kidneys, The, and the Cardio-Vascular System (O.), 129
King, The, and the Consumptives (A.), 247
King Edward's Hospital Fund for London (W.), 409
Kissing the Baby (A.), 145
Labour Colony, A (A.), 417
Labyrinthine Capsule, Deafness from Rarefaction of (P.), 361
"Lachnanthes Cure" of Consumption, The, 234
Lack of Sanitary Organisation in London, The (A.), 27
Ladies on Hospital Boards (W.), 39
Lady House Surgeons, 110
Lady House-Surgeons in General Hospitals (W.), 156
Lager Beer, The Medicinal Uses of (A.), 61
Laminectomy in Fracture of the Spine (C.), 149
Lamps, New, for the Light Treatment (O.), 13
Lancashire County Asylums (W.), 241, 315
Laryngeal Neoplasms (P.), 273
Larynx, Tuberculosis of the (P.), 272
Lead-poisoning in a Child (P.), 219
Legislation of 1902, The, 211
Leper Homes, 36
Lepers in England (A.), 77
Leprosy, 160
Leprosy, The Fish Theory of (O.), 251
Leucæmia (P.), 134
Leucoplakia of the Vulva and Cancer (P.), 326
Lichen (P.), 53
Life-saving in the London Streets (W.), 224
Light and X Rays, Treatment by (O.), 13
London Fogs, 76
London, The Health of (O.), 217
London Hospital, The (W.), 410
London School of Tropical Medicine (W.), 71
London University, The Incubus of the (A), 179
London Water Supply, The (A.), 335
Lungs, Disease of the (P.), 359, 375
Lupus, The Scraping of (C.), 13
Lymph, Official (A.), 283
Lymphadenoma (P.), 186
Macclesfield, The Difficulty at (A.), 179
Macclesfield Infirmary Dispute, The (W.), 242
Man in the Crystal Urn, The (A.), 161
Manual Training in the Prison (A.), 353
Martyr to Duty, A (W.) 243
Massage in Simple Fractures of the Leg (C.), 81
Matlock House Hydropathic Establishment (W.), 259
Mastoid, Auscultation of the (P.), 375
Mastoid Operations, The Technique of (P.), 374
Mastoiditis (P.), 361
Maxillary Antrum, Diseases of the (P.), 306
Mediæval Hospitals in France (W.), 241
Medical Faculty, The, and the Academic Council of the University of London (A.), 353
Medical Herbalists, The Status of (A.), 127
Medical Psychology, 230
Medical Reform (A.), 77
Medical Year, The (O.), 233
Medicine, Progress in (P.), 389, 407
Medicine and Sociology, 144
Melancholia and the Vesanic Processes (P.), 255
Melancholia with Delusions, Pathogenesis of (P.), 290
Meningitis (P.), 133
Meningitis, Acute Delirium of (P.), 166
Meningitis, Posterior Basic (P.), 219
Mental Fatigue in School Children (P.), 203
Mercurial Lesions (P.), 237
Mercurol (P.), 307
Metropolitan Asylums Board, The Work of the (W.), 240, 258, 277
Metropolitan Hospital Sunday Fund (W.), 105
Middle Ear, Sclerosis of the (P.), 324
Midwives Bill, The, 415
Midwives Bill, Prospects of the (A.), 231
Midwives Board, The Central, 384
Midwives and Infantile Ophthalmia, 300
Milk and Butter, Bacteriology of (P.), 17
"Milk Epidemic" of Scarlet Fever, A (O.), 131
Milk, Raw, in Infantile Atrophy (P.), 203
Model Hospital, A (W.), 207
Modern Sociology, 18, 36, 103, 137, 170, 223, 256, 275, 293, 311, 328, 344, 362
Mount Vernon Hospital, The (W.), 412
Movable Kidney (P.), 99, 422
Muscular Atrophy, Progressive (P.), 150
Music and Mosquitoes (C.), 115
Myasthenia gravis (P.), 150
Myositis, Gonorrhœal (P.), 51
Nasal Polypus (P.), 306
National Hospital, The, 193; (W.), 205; *see pp.* 244, 277
Nauheim Mineral Waters (P.), 252
Neisser's Stain (P.), 83
Neoplasms, Malignant (P.), 289
Nephritis, Interstitial (P.), 85
Nephritis, Peripheral (P.), 151
Nephrolithotomy (P.), 441
Nerves, The Regeneration of divided (O.), 420
Nervous Diseases and Lunacy Laws, 109
Nervous Diseases, Prognosis in (C.), 113
Nervous System in Anæmia, The (C.), 435
Nervous System, Progress in Diseases of the (P.) 132
Neurasthenia, Traumatic (C.), 387
Neurology, Progress in (P.), 118, 133, 150
Neuroses (P.), 119
New Appliances and Things Medical, 17, 35, 53, 101, 119, 135, 169, 187, 203, 255, 273, 291, 309, 377, 390, 408, 441
New Hospital for Women (W.), 413
No Bottles (A.), 433
Noma, Epidemic of (P.), 219
Nose moulded in Paraffin, A (A.), 213
Notes and News, 4, 28, 46, 62, 78, 93, 112, 128, 146, 162, 180, 196, 214, 232, 248, 266, 284, 302, 321, 336, 355, 370, 382, 402, 419, 434
Notifiable Diseases, The Fatality of (C.), 113, 386
Obesity, 317
Obstetrical Society of London, The (O.), 49
Obstetrics, Progress in (P.), 389, 405
Œdema Glottidis (P.), 272
Œsophagus, Stricture of the (O.), 81
"One Portal" Panacea, The, 26
Oöphorectomy in Cancer (O.), 251
Oöphorectomy, Dr. Roger Williams on (C.), 269
Operation's Good Repute, An (A.), 301
Ophthalmia Neonatorum, Bacteriology of (P.), 377
Ophthalmology, Progress in (P.), 168, 185
Otitic Temporo-sphenoidal Abscess (P.), 375
Otitis media, Acute (P.), 307
Otology (P.), 307, 324, 360, 374
Otorrhœa, Treatment (P.), 324
Our Children, 159
Ovarian Pregnancy (P.), 406
Ozæna (P.), 35
Paralysis, General (P.), 133
Parasites, Harmless (O.), 115
Payment for Revaccination (A.), 369
Pearls (A.), 231
Pediatrics, Progress in (P.), 202, 218
Pelvic Bones, Movements of, during Symphyseotomy (P.), 389
Pericardium, Tuberculosis of the (P.), 308
Peristalsis, Intestinal (O.), 235
Peritonitis, Pneumococic, in Children (P.), 219
Peritonitis, Tuberculous (P.), 340
Peritonsilitis (P.), 238
Pince-nez, An Evil Consequence of (O.), 437
Phthisiophobia, 299
Plague and its Prevention (P.), 14
Plague, The Recrudescence of (A.), 93
Pleurisy with Appendicitis, The Association of (O.), 148
Pneumomycosis (P.), 375
Polypoid Disease of the Nose, Treatment of (O.), 405
Popular Medicine, 400
Practical Departments (W.), 23, 57, 73, 123, 191, 313, 329
Pregnancy, Uncontrollable Vomiting of (P.), 99
Prince and the Hospitals, The, 339
Prince of Wales, The, at the St. Bartholomew's Hospital (W.), 188
Prince of Wales's Hospital Fund, 121, 226
Prodromal Symptoms (P.), 271
Professional Reputation, 43
Prostate, Treatment of Enlarged (O.), 147, 355
Prostate, The, and Dr. Freyer's Operation (O.), 356
Protargol in Gonorrhœa (P.), 34
Psychiatry, Progress in (P.), 50, 66, 151, 166, 255, 271, 290
Puerperal Eclampsia (P.), 98
Puerperal Infection (P.), 99
Puerperal Insanity, its Ætiology and Classification (P.), 50
Puerperal Insanity, Duration of (P.), 66
Pyæmia, Otogenous (P.), 375
Pylorectomy, The Sequel in (O.), 268
Quacks, Our Friends the (A.), 77
Quinine, The Subcutaneous Injection of (O.), 373
Railway Lavatories (W.), 123
Rangoon Lunatic Asylum, 55
Rectal Feeding (O.), 79
Renal Calculi, Accidental Dislodgement of (O.), 199
Renal Disease, The Diagnosis of (P.) 117
Renal Tension, Surgical Treatment of (P.), 118
Reservoirs and Rivers (A.), 3
Rest Cure, The (C.), 13
Retinitis, Albuminuric, Prognosis in (O.), 217
Re-vaccination (C.), 303, 321
Reversed Test Types (C.), 97
Rheumatism (P.), 369, 407
Rickets Fœtal or Congenital (P.), 202
Ringworm in Schools (A.), 93
Ringworm, Obstinate, Treatment of (O.), 115
Roentgen Ray Work, Progress in (P.), 237
Roof Gardens (W.), 140
Royal Army Medical Corps, 91
Royal Chest Hospital (W.), 429
Royal Dental Hospital of London (W.), 428
Royal Eye Hospital (W.), 447
Royal Sea-Bathing Hospital, Margate (W.), 445
Royal Westminster Ophthalmic Hospital (W.), 414
Royston Isolation Hospital (W.), 21
St. Mary's Hospital, Paddington (W.), 429, 446
Salt Pack in Rheumatic Gout (O.), 324
Sanatoria, Rate-supported (A.), 145
Sanatorium Treatment, Essentials of (O.), 216
Sanitary Dealings with Subject Races, Our (A.), 11[illegible]
Sanitation in India, 311, 329
Scapulo-Humeral Reflex (P.), 118
Scarlet Fever, The Blood in (P.), 187
School for the Indigent Blind (W.), 139
Scoliosis (O.), 6
Scurvy, Infantile, and "Sterilised" Milk (O.), 372
Sea Voyage for Inebriety (O.), 80
Seborrhœa (P.), 52
Self-Drugging by the Public, 1
Senility, Premature (O.), 285
Septal Deflections (P.), 306
Sewage and its Treatment (P.), 83, 423
Sewer Air (A.), 353
Shock, The Prevention of, during Operations (O.) 65
Slums and Manslaughter (A.), 401
Small-pox (P.), 342
Small-pox, Diagnosis of (O.), 29, 318
Small-pox. How does Small-pox Spread? 92
Small-pox, The Fight against (W.), 443
Small-pox, The Notification of (A.), 161
Small-pox Panic, 334
Small-pox and the Seasons (A.), 45
Small pox and Vaccination (A.), 417
Special Schools under the London School Board, 344, 362
State Medicine, Progress in (P.), 14, 83, 220, 238, 341, 423
Stethoscope, Binaural, Sins of the (O.), 356

180507

Stomach, Acute Dilatation of the (C.), 79
Stomach Tube, Passing the (C.), 267
Storage of Thames Water, The (A.), 61
Stourfield Park Sanatorium, Pokesdown, Bournemouth, 55
Strabismus (P.), 185
Strain, Heart (P.), 32
Student of Medicine, The, 24, 42, 58, 74, 90, 108, 124, 142, 158, 175, 192, 210, 228, 244, 261, 280, 298, 315, 332, 350, 366, 414, 448
Study of the Child, The, 223
Sudden Death in Infancy and Childhood (P.), 202
Suppuration, Fœtid and Gangrenous (P.), 271
Suprarenal Extract as a Hæmostatic (C.), 323
Surgery, Cerebral (P.), 200
Surgery, Genito-Urinary (P.), 153, 167
Surgery of the Large Intestine (C.), 322
Surgery, Progress in (P.), 33, 51, 99, 117, 153, 167, 324, 360, 374, 422, 440
Surgery, Renal (P.), 99
Susceptibility (A.), 213
Susceptibility to Re-Vaccination (C.), 268
Syphilis and Heart Disease (P.) 33
Syphilis, Primary of the Conjunctiva (P.), 52
Syphilis, Some Points in the Symptomatology of (P.), 52
Syphilis in the Well-to-do (P.), 52
Syphilitic Brain Disease (C.), 164
Tabes Mesenterica (C.), 304
Tea-kettle, The Policy of the (A.), 127
Telephones, 177
Tendo Achillis, Rupture of the (C.), 287
Tetanus (P.), 357
Throat and Nose, Diseases of the (P.), 237, 253, 272, 289, 306
Thrombosis, Venous (C.), 80
Thyroid, Progress in Diseases of the (P.), 68
Thyroid Gland, Derangements of the (C.), 164
Tight Lacing, Cycling and Nephroptosis (C.), 198
Tinnitus Aurium, Effects of (P.), 361
Tobacco Heart (P.), 32
Tonsillitis, Membranous (P.), 270
Tonsils, Hypertrophy of the (P.), 237
Tracheal Tug (P.), 309
Transition to Winter, The, 25
Tropical Diseases (P.), 67
Tubercle Bacillus, The (P.), 270
Tuberculosis (P.), 238
Tuberculosis, Etiology and Pathology (P.), 359
Tuberculosis, Human and Bovine (P.), 16
Tuberculosis among the Jews (C.), 286 and *see p.* 331
Tuberculosis Treatment, 360
Tuberculosis, Treatment of, with Urea (C.), 182
Tuberculous Children in Schools (A.), 401
Tuberculous Glands in the Neck, Operation for (C.), 32
Tumours complicating Pregnancy and Labour (P.), 98
Tumours of the Frontal Lobe of the Brain (P.), 150
Ulcer of the Cornea, Chronic Serpiginous (C.), 131
Ulcers (P.), 340
Universal Military Service (A.), 247
University, at last, A Teaching (C.), 305
University Matriculation, 368
Unprotected Infants (A.), 433
Urethra, Irrigation of the (C.), 165
Urethroscopy (P.), 167
Urine, Tubercle Infection of (P.), 82
Urology (P.), 116
Uterine Fibroids, Operations on (C.), 32
Uterine Fibroids. The Treatment of (P.), 325
Uterine Retraction (P.), 405
Uterus, Cancer of the (P.), 325
Uterus, Obstinate Prolapse of the (C.), 31
Uterus, Retro-displacement of the, Surgical Treatment of (P.), 326
Uterus, Rupture of the (P.), 390
Vaccinate, How to (C.) 287
Vaccination: What is Efficient Vaccination? (A.), 195
Vaccination, Modern (C.), 199
Vaccination, Modern Methods of (C.), 337
Vaccination, Public and Private, 125
Vaccination Question, The Other Side of the (C.), 338
Ventilation, Domestic (C.), 81
Ventnor Royal National Hospital for Consumption (W.), 427
Victoria Hospital for Children (W.), 157
Victoria Park Hospital, 429
Victoria University, The Break-up of the (A.), 335
Voluntary System, Modern Developments of the (W.), 21
West Ham Hospital (W.), 412
Whey as a Diet in Typhoid Fever (C.), 96
Widal's Reaction (C.), 437
Women's Restaurants, 256, 275, 293
Workmen's Compensation Act, The (A.), 61
Workmen's Compensation Act and the Testing of Workmen's Sight (P.), 168
Wynnstay Sanatorium, Burgess Hill (W.), 172
X-Rays in Cancer (C.), 183
Yellow Fever (P.), 358
Yellow Fever Experiments in Havana, The, 2
Yellow Fever: Precautions (C.), 215
Yellow Fever at Rio, 59
Youth *versus* Experience (A.), 27
Zymotic Disease in London, 264

### THE SPECIAL SECTION FOR NURSES.

American Red Cross Sisters, 114
American Women and the Buffalo Delegates, 141
Appointments, 19, 38, 52, 62, 76, 88, 105, 113, 130, 139, 158, 168, 178, 193, 207, 221, 233, 245, 259, 274, 284, 296, 310, 324, 336, 350
Army and Indian Nursing Service, The, 5
Baby Typhoid, A, 244
Bananas and how to use them, 112
Bed-Sores, Prevention of, 269
Bed-Sores, Treatment of, 334
"Better Land," The, 19
Beyond the Seas, 14, 151, 215, 241
Book and its Story, A, 22, 64, 101, 144, 170, 183, 234, 287, 311, 352
Cæsarian Section, 126
Chamberlain, Mr., on Nursing, 74
Charing Cross Hospital Nursing Home, 273
Christmas Books, 154
Christmas Distribution of Clothing, Our, 166, 193
Christmas in the Holy Land, 180
Christmas in the Hospitals, 189, 204
Christmas Novelties, 156
Compensation, 60
Congress of Nurses at Buffalo, 8, 30
Cyclone on the Veldt, A, 49
Death in Our Ranks, 38, 104, 114, 137, 207, 231 262, 281, 296, 305, 323, 336
Diary of a Naughty Probationer, 59
District Nurse in America, The, 203
District Nursing, 294
District Nursing in Kimberley, 125
East London Nurses at the Mansion House, 323
Echoes from the Outside World, 18, 36, 53, 63, 77, 90, 102, 118, 131, 143, 159, 169, 182, 194, 312, 325, 338, 351
English Nurse Killed near Mount Lebanon, An, 348
Epileptics, the Care of, 13, 35
Everybody's Opinion, 20, 37, 50, 61, 78, 87, 103, 116, 129, 141, 158, 167, 181, 195, 208, 220, 232, 246, 262, 273, 285, 295, 309, 324, 335, 349
Examination Questions for Nurses, 19, 111
Examination of Nurses at the Royal United Hospital, Bath, 52
Fever Nursing in its Relations to Fever Hospital Administration, 257, 283
Flowers for the Children's Ward, 127
Fragment, A, 229
German Hospital Kitchen, A, 284
Greenwich Guardians and Nurses, 258
Help the Nurses to Help the Sick, 157
Her Christmas Box, 179
Hospital Sketch, A, 15
Hospital for Animals, Bombay, The, 230
Hospitals without Nurses, 97
Krankenhaus in Hamburg, The, 47
Lectures on Gynæcology for Nurses, 188, 226, 240, 268, 292, 317, 344
Lectures to Head Sisters, 153, 217, 231, 243
Lectures to Nurses on Anatomy, 4, 44, 70, 96, 124, 136, 150, 176, 200, 214, 254, 280, 304, 330
Lectures to Nurses on Medicine and Medical Nursing, 28, 58, 84, 110, 164
Maternity Charity and District Nurses' Home at Plaistow, 79
Maternity Nursing Mission, 128
Metropolitan Asylums Board, The, 273
Midwifery Unusual, 111
Midwives Bill, The, 307
Millionaire's Model Ward at Pittsburg, A, 17
New Year and Christmas in the Hospitals, 204
Night Nursing: a Few Words to "Pros," 218
Night Nursing in Mashonaland, 293
Notes on News from the Nursing World, 1, 25, 41, 55, 67, 81, 93, 107, 121, 133, 147, 161, 173, 185, 197, 211, 223, 238, 251, 265, 277, 289, 301, 315, 327, 341
Notes and Queries, 24, 38, 54, 65, 80, 91, 106, 119, 132, 145, 160, 171, 196, 209, 222, 235, 249, 263, 276, 288, 299, 313, 326, 339, 353
Novelties for Nurses, 23, 75, 207, 221, 273, 309, 336
Nurses' Bookshelf, The, 116, 128, 137, 220, 248, 335, 350
Nurses of the Cancer Hospital, The, 177
Nurses for the Concentration Camps, 245
Nurses' Co-operation, The, 138
Nurse's Experience as a Stewardess, A, 348
Nurses in Kilts, 232
Nurses' Never, The, 7, 34, 60, 86
Nurses at the Paddington Green Children's Hospital, 229
Nurses in Public Schools, 115
Nurses of the Royal Berkshire Hospital, 227
Nurses at St. Olave's Infirmary, Rotherhithe, 166
Nurses at St. Pancras Infirmary, The, 282
Nurses of St. Saviour's Union Infirmary, 16
Nurses of the West London Hospital, 85
Nurses of Woolwich Union Infirmary, 307
Nursing in the American Army, 71
Nursing in Bombay Hospitals, 337
Nursing Convalescents, 152
Nursing in Cyprus, 215
Nursing at the Dudley Workhouse Infirmary, 270
Nursing in an Indian Hospital, 255
Nursing in Pietersburg, 202
Nursing at Port Royal, Jamaica, 241
Nursing on the "Princess Christian Hospital Train" at Pretoria, 261
Nursing Question at the Central Poor Law Conference, 331
Nursing at a Small Military Station Hospital, 333
Nursing in South Africa, 29
Nursing in Workhouse Infirmaries, 48
Off-Duty Letter, 275, 286, 298
One Easter Eve, 345
Position, The Importance of, in Accident and Disease, 297
Presentations, 38, 60, 73, 89, 100, 114, 130, 153, 168, 195, 207, 221, 233, 268, 285, 296, 323, 336, 350
Queen Victoria's Jubilee Institute for Nurses, 192, 260
Rathbone, The late Mr. William, of Liverpool, 318
Royal British Nurses' Association, The, 75, 137, 229
Royal British Nurses' Bazaar, 272
Royal National Pension Fund, A Nurse on the, 45
Scheme for Affiliated Hospital Nursing, 347
Sheppey Workhouse Infirmary, 246
Shoreditch Nurses in Harley Street, 323
Sick-Room Cookery, 201, 216, 229, 242, 258
Six Months' Private Work, 308, 332, 346
Small-pox, Appearance and Nursing of, 165
Souvenir of Marlborough House, 100, 167
Thermometer, How to Use a Clinical, 113
Three Magazines, 180
Travel Notes, 40, 66, 92, 120, 146, 172, 210, 236, 250, 264, 285, 300, 314, 340, 354
Undressing of Patients Suffering from Fractures, The, 219
Victoria Nurses' Home, Northampton, Opening of the, 167
Visit to an Indian Nursing Home, 73
Wants and Workers, 52, 88, 104, 168, 193, 219, 233, 240, 285, 305
Wardwork, Necessary and Unnecessary, 98
Weddings in Our Ranks, 192
Where to Go, 89, 104, 115, 125, 168, 233, 241, 260
"Word of Welcome," A, 305
Workhouse Infirmary Nursing, 130
Workhouse Nurses, The Scarcity of, 319

Printed by SPOTTISWOODE & CO. LTD., New Street Square, E.C.; and Published by the Proprietors, THE SCIENTIFIC PRESS, LIMITED, at 28 & 29 Southampton Street, Strand, London, W.C.

### NOTICE TO CORRESPONDENTS.

All MSS., Letters, Books for Review, and other matters intended for the Editor should be addressed The Editor, "The Hospital," The Hospital Building, 28 & 29 Southampton Street, Strand, W.C.

The Editor cannot undertake to return rejected MS., even when accompanied by stamped directed envelope.

### Notice to Advertisers and Subscribers.

All Advertisements, Orders for Copies of The Hospital, and Business Communications should be addressed to **THE MANAGER (Not to the Editor)**, The Hospital, 28 & 29 Southampton Street, Strand, London, W.C.

The Cost of Subscription, post free, is as follows:—Per week, 2½d.; per quarter, 2s. 8d.; per half-year, 5s. 3d.; per annum, 10s. 6d. Foreign:—Per annum, 15s. 2d., payable, in all cases, in advance.

**NOTICE.—Vol. XXX. of "The Hospital" (April 1901, to September 1901), in handsome cloth binding will shortly be on sale at the Office of this Journal, price, 7s. 6d. Cases for binding the Numbers contained in this Volume 1s. 6d. Index to Vol. XXX., 2½d., post free.**

**The Monthly Part for October is now ready. Price 6d., by post 8d.**

### NOTES AND NEWS.

### THE STUDENT OF MEDICINE.

### APPOINTMENTS.

**H. H. H. Addenbrooke**, L.R.C.P., L.R.C.S.Edin., appointed District Medical Officer of the Solihull Union. **Robert W. Beesley**, M.D., C.M.Edin., M.R.C.P.Edin., M.R.C.S.Eng., L.R.C.P.Lond., appointed Honorary Medical Officer to the Bolton Infirmary and Dispensary. **C. Branwell**, M.R.C.S., L.R.C.P.Lond., appointed Medical Officer for the Fifth District of the Penzance Union. **William H. Bryce**, M.B., C.M.Edin., appointed Honorary Medical Officer to the Bolton Infirmary and Dispensary. **Gordon Calthorpe**, M.B., B.C.Camb., appointed Medical Officer of Health for the Wells Urban and Port Sanitary District. **J. L. B. Dixon**, M.D., B.S.Vict., appointed Assistant Medical Officer of Health of the City of Brisbane, Queensland. **H. A. H. Gilmer**, M.B., Ch.B.Edin., appointed House-Surgeon to the David Lewis Northern Hospital, Liverpool. **A. W. Hill**, M.D., appointed Ophthalmic Surgeon to the Adelaide Hospital. **L. N. Hoystead**, M.R.C.S.Eng., L.R.C.P.Lond., appointed Officer of Health for Hampden, Victoria. **E. S. Humphrey**, M.R.C.S.Eng., L.R.C.P.Lond., appointed Medical Officer at Southern Cross, Western Australia. **H. Johnson**, L.R.C.P., L.R.C.S.Edin., appointed Medical Officer for the South District of the Lincoln Union. **William Crosby Johnson**, M.B., Ch.B.Vict., appointed Junior House-Surgeon to the Salford Royal Hospital. **Joseph Edward Judson**, M.R.C.S.Eng., L.R.C.P.Lond., appointed Senior Resident Medical Officer of the Manchester Workhouse, Crumpsall. **R. E. Lauder**, F.R.C.S., L.R.C.P.Edin., D.P.H., appointed Medical Officer of Health for the Borough and Port of Southampton. **E. R. MacDonnell**, L.R.C.P., L.R.C.S.Edin., appointed Medical Officer for the Lifton District of the Tavistock Union. **F. D. S. Mackenzie**, M.B., Ch.B.Edin., appointed Assistant House-Surgeon to the David Lewis Northern Hospital, Liverpool. **J. F. Matthews**, M.R.C.S.Eng., appointed Officer of Health for Portland E., Victoria. **B. J. Mayne**, M.R.C.S., L.R.C.P.Lond., appointed Medical Officer for the Illogan District of the Redruth Union. **R. S. Rogers**, M.A., M.D., appointed Honorary Physician to the Adelaide Hospital. **B. C. Scott**, M.R.C.S.Eng., appointed Assistant Medical Officer to the West Bromwich Union Workhouse

